# Prevalence of hepatitis B virus and hepatitis C virus infection in patients with systemic lupus erythematosus: a systematic review and meta-analysis

**DOI:** 10.18632/oncotarget.22261

**Published:** 2017-11-01

**Authors:** Sen Wang, Yuxin Chen, Xuejing Xu, Wei Hu, Han Shen, Junhao Chen

**Affiliations:** ^1^ Department of Laboratory Medicine, Nanjing Drum Tower Hospital, Nanjing University Medical School, Nanjing 210008, Jiangsu Province, China

**Keywords:** hepatitis B virus, hepatitis C virus, systemic lupus erythematosus, meta-analysis, systematic review

## Abstract

We attempted to explore the prevalence of HBV and HCV infections in patients with systemic lupus erythematous (SLE) via a systematic review. Articles published before June 2017 and, related to prevalence rates for HBV and HCV infection in SLE patient were identified in PubMed, Embase, CNKI, and Wanfang databases. Based on these searches 22 studies were selected for further analysis. The OR of HBsAg positive rate in SLE patients compared with control population was 0.28, with significant heterogeneity identified among the studies (*I*^2^ = 92%, *P* < 0.00001). Following exclusion of one study, the adjusted OR of HBsAg in patients with SLE was 0.24, and no significant heterogeneity was observed (*I*^2^ = 32%, *P* = 0.15). The adjusted OR of HBcAb positive rate in SLE patients compared with control population was 0.40, with no significant heterogeneity between studies (*I*^2^ = 0%, *P* = 0.56). The risk of having HCV infection by SLE patients was higher compared with the control subjects (OR = 2.91). In conclusion, this meta-analysis suggested that SLE might exert a role of protection against HBV but not for HCV infection. Further epidemiological and experimental studies are necessary to explore the role and mechanisms by which SLE affects HBV/HCV infections.

## INTRODUCTION

Hepatitis B virus (HBV) and hepatitis C virus (HCV) are the two most common hepatotropic viruses responsible for majority of acute and chronic hepatitis worldwide [1]. Over 350 million individuals chronically infected with HBV are at high risk of developing liver cirrhosis and hepatocellular carcinoma (HCC) [2]. HBV is a small hepatotropic DNA virus. Serological markers for HBV infection include HBsAg, anti-HBs, HBeAg, anti-HBe, and anti-HBc. HBsAg is the hallmark of infection while HBcAb is an indicator of past or present hepatitis B infection [3]. The prevalence of HBV infection varies markedly throughout the world. 45% of HBV-infected individuals live in highly endemic areas, which include China, Southeast Asia, sub-Saharan Africa and the Amazon Basin [4, 5]. Chronic infection with HCV is another major risk factor for the development of HCC worldwide. Unlike HBV, HCV is a positive-sense single-stranded RNA virus of the family *Flaviviridae* [6]. The highest prevalence of HCV has been reported in Africa and the Middle East, with a lower prevalence in the Americas, Australia and Northern and Western Europe [7].

Systemic lupus erythematous (SLE) is a classic systemic autoimmune diseases characterized by chronic inflammation, autoantibodies production, complement activation and immune-complex deposition, resulting in tissue and organ damage. SLE is occurring predominantly in young women with the female: male ratio of 10:1. Overall, the incidence of SLE is higher in African, American and Asian populations than in Caucasians [8]. The cause of SLE is not fully understood, but it is believe to involve hormonal, environmental, genetic and immunological factors [9].

Autoimmune disease is closely related to viral infection since it can be induced or triggered by viruses. For example, EBV was found to be associated with SLE and RA [10]. In contrast, some viral infections can also protect individuals from specific autoimmune diseases. CMV and EBV infection were shown to play a protective role in type 1 diabetes [11, 12]. Although the potential association between HBV and SLE has been studied for decades it still remains controversial. HCV infection is associated with many autoimmune processes [13], but the association between HCV infection and SLE disease has not been clearly established. Therefore, the current study was designed to evaluate the prevalence of hepatitis virus infection among SLE patients through meta-analysis of published studies.

## RESULTS

### Characteristics of the included studies

**Figure 1:**
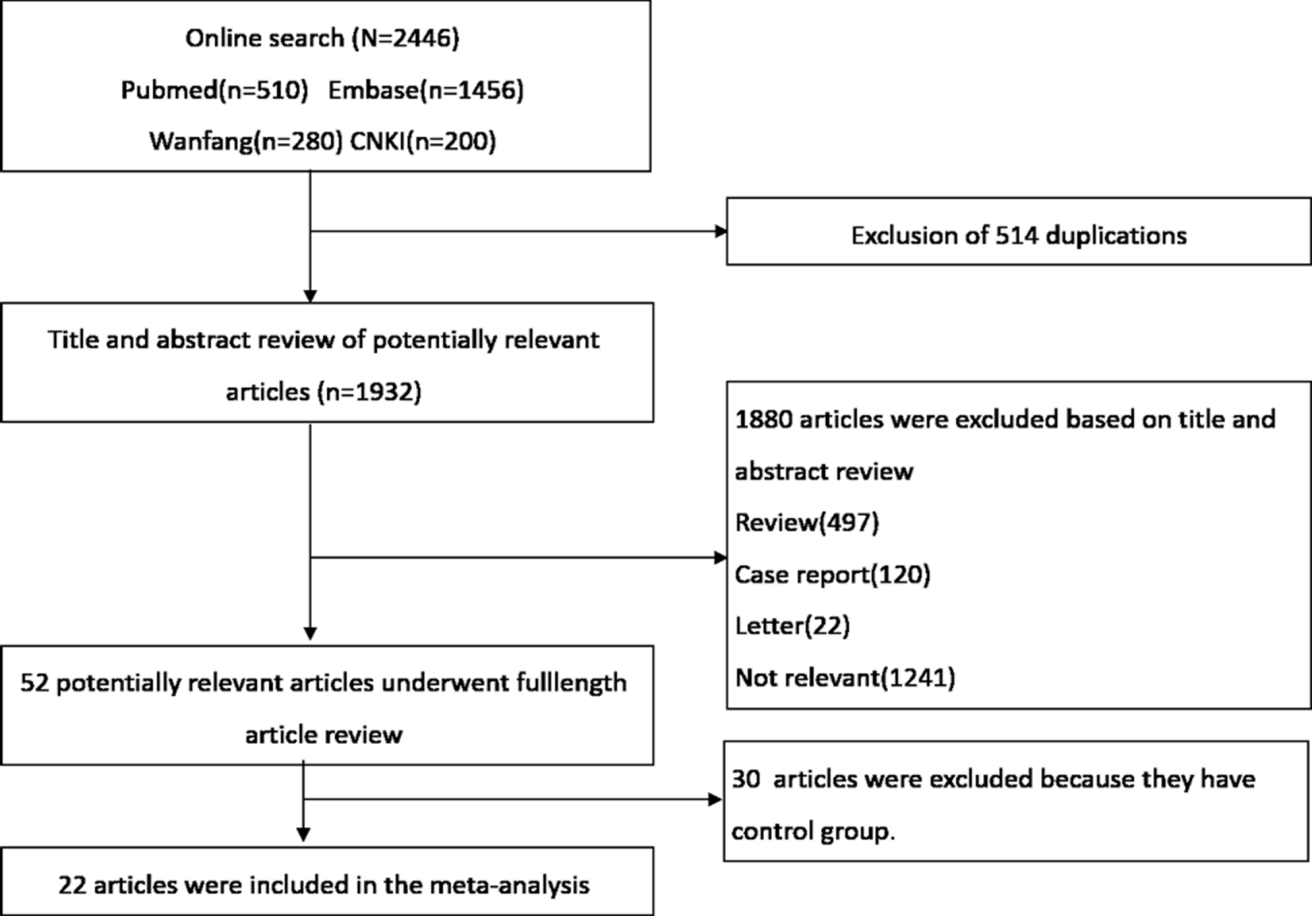
Flowchart depicting the selection of studies for the meta-analysis

A flow diagram of studies selection is shown in Figure 1. A total of 2446 articles met the defined search criteria. Further screen of their titles and abstracts identified the potentially relevant articles. Finally, 22 studies were selected for the meta-analysis [14–33].

As listed in Table 1, 11 case-control studies, published between 1997 and 2016, were identified to analyze HBV infection state using HBsAg. All of the 11 studies were from the Asian. Of those, 8 studies were from China, one from China Taiwan, one from Japan and one from Israel. 4 studies were retrospective design, and 7 were prospective. Altogether, 127 cases of HBV infection in 9333 SLE patients were reported. Among 203329 control individuals, 16392 cases of HBV infection were identified. Controls included healthy subjects in 4 studies, non-SLE inpatients in another 4 studies, and 3 studies used rheumatoid arthritis (RA) patients as controls.

**Table 1:** Characteristics of included studies (HBsAg)

Study	Publication year	Country	Type of study	SLE patients(n)	Controls(n)	Control description	Matching and adjustments	HBsAg detection	NOS score	Reference
Wang F, et al.,	2016	China	Prospective	346	12779	Healthy subjects	Age and sex	NA	6	14
Sui M, et al.	2014	China	Retrospective	155	3122	Non-autoimmune population	Age and sex	ELISA/CMIA	7	15
Watanabe R, et al.	2014	Japan	Retrospective	1128	3580	RA	NA	NA	6	16
Zhao J, et al.	2010	China	Retrospective	859	155222	Non-SLE inpatients	Age and sex	NA	8	17
Zheng BR, et al.	2010	China	Retrospective	379	209	Physical examination takers	NA	NA	5	18
Lu CL, et al.	1997	Taiwan, China	Prospective	173	692	Healthy subjects	Age and sex	Radioimmunoassay kits	8	19
Cao YX, et al.	2002	China	Prospective	131	582	Healthy controls	Age and sex	Elisa	6	20
Wang S, et al.	2016	China	Retrospective	866	1795	Healthy controls	Age and sex	Elisa	7	21
Wu CX, et al.	2005	China	Retrospective	176	194	RA	Age and sex	NA	5	22
Qiu N, et al.	2011	China	Prospective	102	64	Non-SLE inpatients	NA	Elisa	5	23
Gendelman O, et al.	2016	Israel	Retrospective	5018	25090	Non-SLE inpatients	Age and sex	NA	6	24

Table 2 showed the 7 studies published between 1997 and 2016, which analyzed the past or present hepatitis B infection using HBcAb status. Among these studies, 3 studies were from China, 2 studies were from Israel, one from China Taiwan and one from Japan. 4 studies selected controls from healthy subjects, 1 study used non-SLE inpatients and 2 studies used rheumatoid arthritis (RA) patients as controls.

**Table 2:** Characteristics of included studies (HBcAb)

Study	Publication year	Country	Type of study	SLE patients(n)	Controls(n)	Control description	Matching and adjustments	HBcAb detection	NOS score	Reference
Wang F, et al.,	2016	China	Prospective	346	12779	Healthy subjects	Age and sex	NA	6	14
Watanabe R, et al.	2014	Japan	Retrospective	248	703	RA	NA	NA	6	16
Ram M, et al.	2008	Israel	Prospective	117	140	Individuals without inflammatory, autoimmune disease or history of chronic infection	Age and sex	Elisa	7	25
Berkun Y, et al.	2009	Israel	Prospective	120	140	Without known inflammatory or autoimmune disease	Age and sex	BioPlex 2200 Multiplexed Immunoassay method	7	26
Lu CL, et al.	1997	Taiwan, China	Prospective	173	222	Healthy subjects	Age and sex	Radioimmunoassay kits	8	19
Wu CX, et al.	2015	China	Retrospective	176	194	RA	Age and sex	NA	5	22
Qiu N, et al.	2001	China	Prospective	102	64	Non-SLE inpatients	NA	Elisa	5	23

9 studies published, between 2000 and 2017, were included to analyze the prevalence of HCV infection among SLE patients. All of the 9 studies were conducted either in Asia or South America. 7 studies were retrospective studies and 2 were prospective. The quality of included studies ranged from moderate to good, varying from six to eight points, based on NOS scoring system. The detail characteristics of the included studies are listed in Table 3.

**Table 3:** Characteristics of included studies (HCV)

Study	Publication year	Country	Type of study	SLE patients (*n*)	Controls (*n*)	Control description	Matching and adjustments	HCV detection	NOS score	Reference
Feng, H., et al.	2006	China	Prospective	92	58	Normal control people	Age and sex	PCR	6	27
Mahroum, N., et al.	2017	Israel	Retrospective	5018	25090	Controls without SLE	Age and sex	NA	6	28
Ramos-Casals, M., et al.	2000	Spain	Prospective	134	200	Volunteer blood donors	Age and sex	Elisa, RIBA, PCR	6	29
Agmon-Levin, N., et al.	2009	Israel	Prospective	108	236	Healthy controls	Age and sex	Elisa	7	30
Mercado, U., et al.	2005	México	Prospective	110	300	Blood donors	NA	Elisa, RIBA, PCR	6	31
Wang,S., et al.	2016	China	Retrospective	844	1348	Blood donors	Age and sex	Elisa	7	21
Berkun, Y., et al.	2009	Israel	Prospective	120	140	Non-autoimmune controls	Age and sex	BioPlex 2200 Multiplexed Immunoassay method	7	26
Mowla, K. and E. Hajiani,	2008	Iran	Prospective	109	110	Healthy donors	Age and sex	Elisa, PCR	8	32
Costa, C.A., et al.	2002	Brazil	Prospective	91	8867	Volunteer blood donors	Age and sex	Elisa, PCR	6	33

### The prevalence of HBV infection in patients with SLE

**Figure 2:**
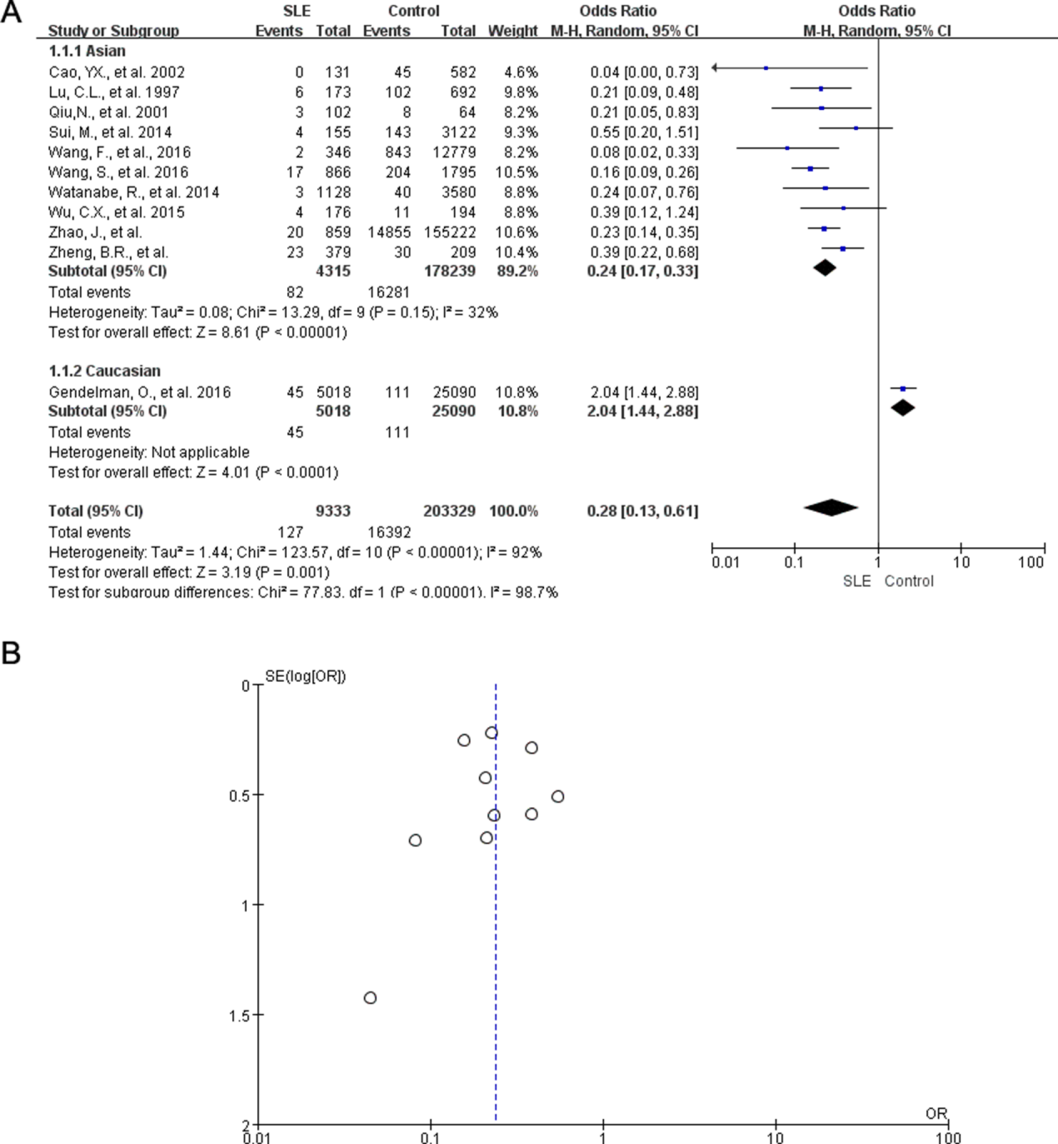
Meta-analysis of HBsAg positivity rate in SLE patients and controls (**A**) Forest plot of the meta-analysis for the HBsAg positive rate in patients with SLE versus controls, OR, odds ratio; CI, confidence Interval. (**B**) Funnel plots showing lack of bias in 11 studies measuring the rate of HBsAg positivity in SLE patients and controls.

We identified 11 case-control results that associated SLE with HBsAg positivity indicative of HBV infection. Of those, 10 studies from Asia reported relatively lower rate of HBsAg positivity in patients diagnosed with SLE compared with the control population, whereas one study from Israel did not support this observation [24]. In a meta-analysis of these studies, the pooled odds ratio was (OR: 0.28; 95% CI: 0.13, 0.61) in a random effect model for HBsAg positive rate in SLE patients compared with control population. However, we detected significant heterogeneity among the studies (*I^2^* = 92%, *P* < 0.00001), which might be due to the study from Israel. If this study was further excluded, the adjusted OR of HBsAg in patients with SLE was 0.24 (95% CI: 0.17, 0.33). No significant heterogeneity was observed after adjustment (*I^2^* = 32%, *P* = 0.15) (Figure 2A). The funnel plot and egger's test were used to assess the publication bias of the included studies. The funnel plot was symmetric (Figure 2B) and the egger's test showed no significant publication bias (*P* = 0.869).

**Figure 3:**
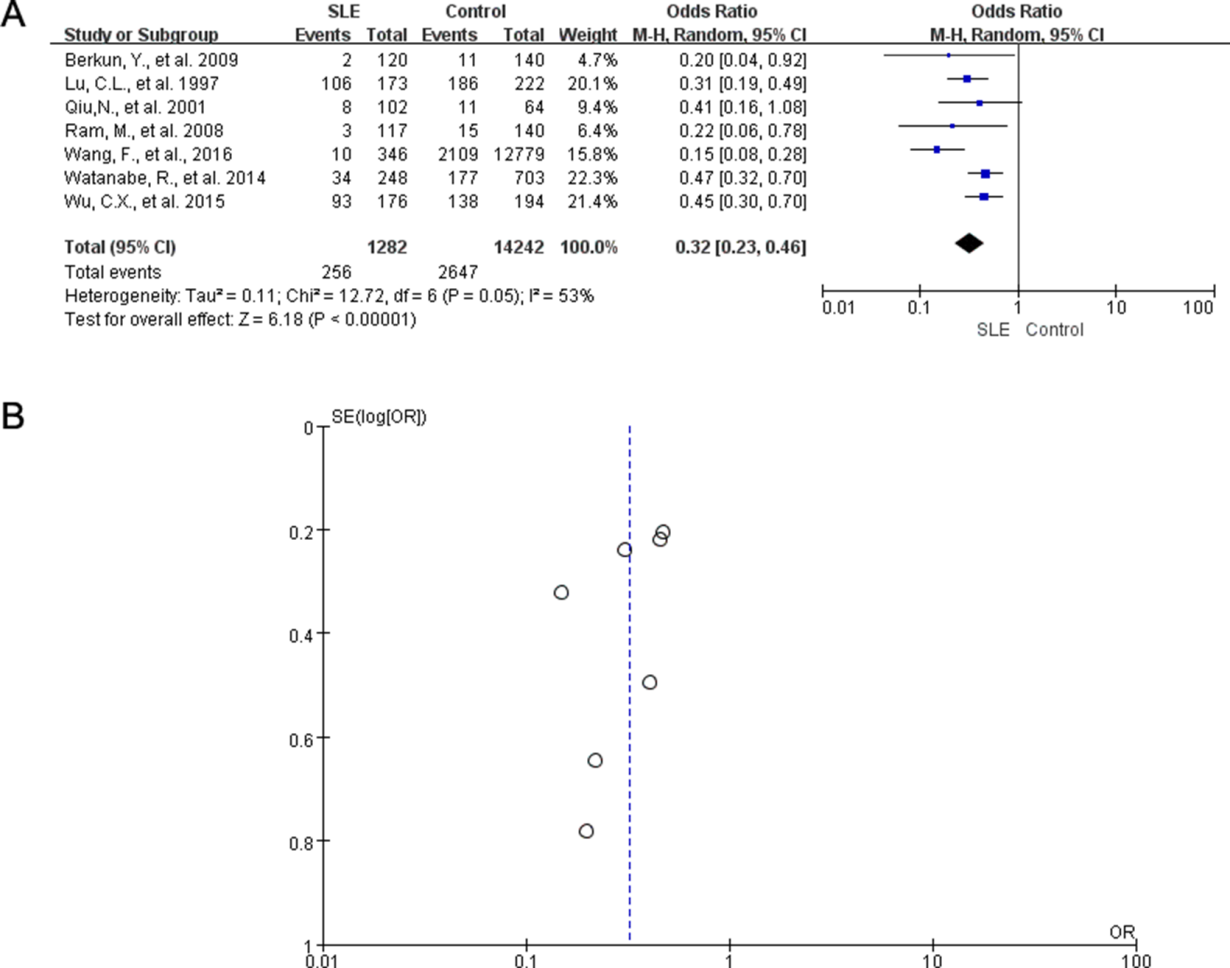
Meta-analysis comparing the rate of HBcAb positivity in SLE patients and controls (**A**) Forest plot shows the HBcAb positive rate in patients with SLE versus controls, OR, odds ratio; CI, confidence Interval. (**B**) Funnel plots showed no publication bias in 7 studies measuring HBcAb positive rate in SLE patients and controls.

Moreover, we conducted additional meta-analysis to evaluate the infection status of HBV in SLE patients using the HBcAb positive rate. For this, 7 studies with 1282 participants were included in pooled analysis. The risk of HBcAb-positive rate in patients with SLE was 3.13-fold lower (pooled OR 0.32, 95% CI 0.23–0.46) than that in the control population. There was a moderate heterogeneity between studies with an *I^2^* of 53% (*P* = 0.05) (Figure 3A). If the study from Wang, F., et al. 2016 was excluded, the adjusted OR of HBsAg in patients with SLE was 0.4 (95% CI: 0.31, 0.50). No significant heterogeneity was observed after adjustment (*I^2^* = 0%, *P* = 0.56) (Supplementary Table 1). The funnel plot was symmetric (Figure 3B) and the egger's test showed no significant publication bias (*P* = 0.145).

### The prevalence of HCV infection in patients with SLE

**Figure 4:**
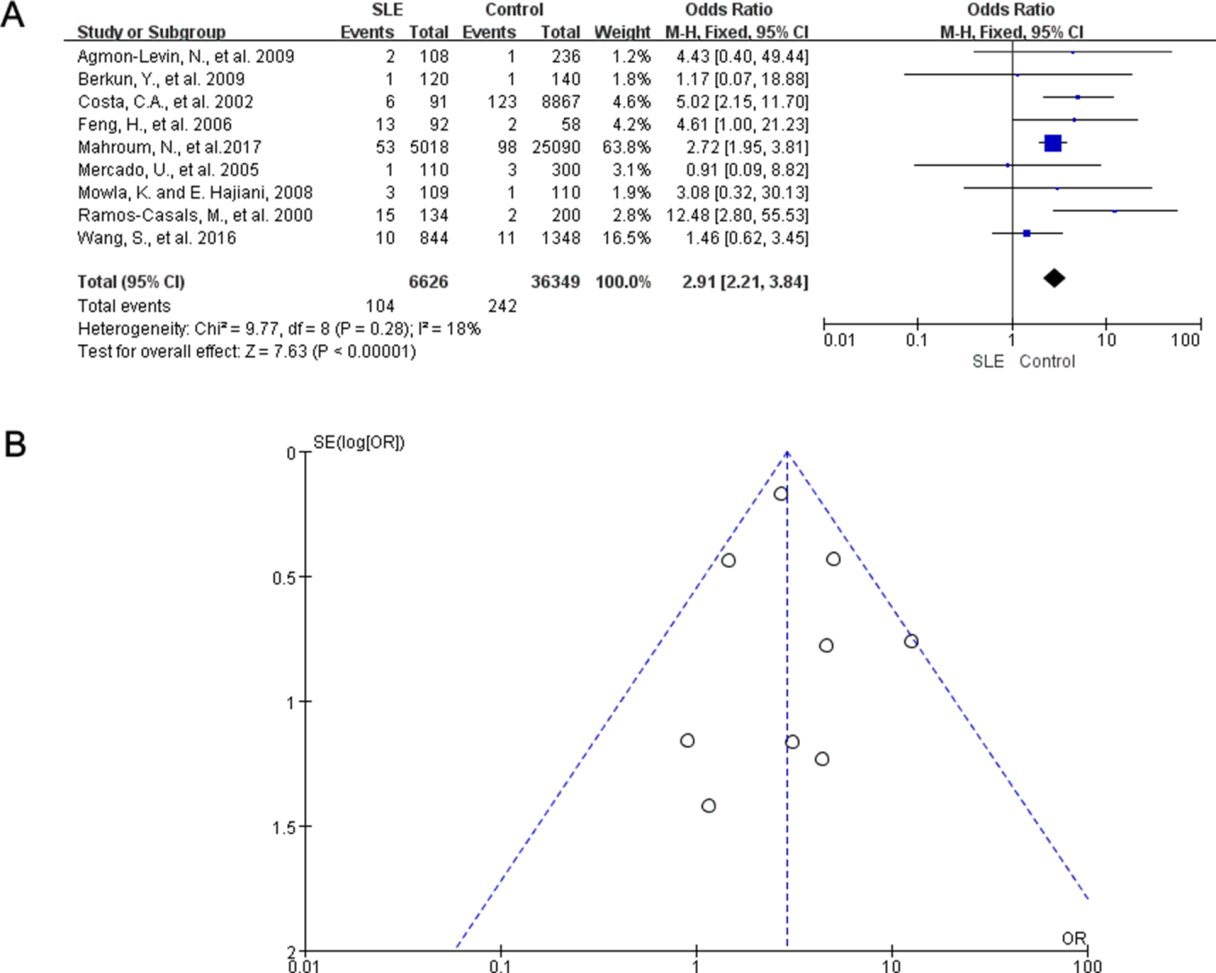
Meta-analysis of the prevalence rate for HCV in SLE patients and controls (**A**) Forest plot showing the prevalence of HCV infection in patients with SLE versus controls. OR, odds ratio; CI, confidence Interval. (**B**) Funnel plots showed no publication bias in 9 studies examining HCV prevalence rate in SLE patients and controls.

We also conducted a meta-analysis to evaluate the risk of HCV infection in SLE patients. 9 case control studies reported the prevalence of HCV infection in SLE patients. Of those, 8 studies showed that the HCV positive rate was higher in SLE patients compared with the control population. In the meta-analysis of all 9 studies, the pooled OR was 2.91 (95% CI: 2.21, 3.84) in a fixed-effects model (Figure 4A), There was no statistically significant heterogeneity among studies (*I^2^* = 18%; *P* = 0.28). The subgroup analysis showed no significant change in both the PCR group and non-PCR group (Supplementary Figure 1). Omission of any single study did not significantly change the overall OR (Supplementary Table 2). No evident asymmetry of plots was seen in the funnel plots (Figure 4B) and the Egger's test showed no significant publication bias (*P* = 0.512).

## DISCUSSION

The infection of HBV and HCV has posed a major public-health problem worldwide and is the major cause leading to chronic liver disease. Ineffective antiviral immune responses have been implicated as the critical factors leading to development of chronic HBV and HCV infections [34]. Although, viral infections and autoimmunity are closely associated their association often remains controversial. For example, some studies proposed that HBV or HCV infection represent a triggering factor for development of systemic autoimmune diseases [13, 34]. In contrast, it has also been suggested that HBV infection can afford a protection against development of autoimmune disorders [11, 35]. SLE is a classical autoimmune disease, which similarly to HBV affects many people worldwide, however the relation between SLE and hepatitis virus infection remains unknown. Here, we present a meta-analysis to explore the prevalence of HBV or HCV in SLE patients. The results of our meta-analysis point to a low prevalence of HBV infection in patients with SLE in highly endemic areas. The risk of HBsAg positive rate in SLE patients was lower when compared with the control group, suggesting a protective role of pro-inflammatory environment in SLE against HBV infection. This is in contrast with HCV infections, which manifest high prevalence in SLE patients compared to controls.

In this meta-analysis, the pooled OR was 0.28 for HBsAg positive rate in SLE patients compared with control population, however a significant heterogeneity was observed among the studies. Particularly, a sole study conducted in Israel revealed a different conclusion. Upon exclusion of this study, the adjusted OR of HBsAg in patients with SLE was 0.21, and no significant heterogeneity was then observed. However, our analyses suggest that a positive rate of HBsAg in SLE patients may vary among different endemic regions. Of interest, in highly endemic Asian areas, HBsAg positive rate was lower in patients with SLE than that of the control group. Moreover, to evaluate the prevalence of HBV infection in SLE patients we performed additional meta-analysis in which the level of anti-HBcAb was analysed. The presence of anti-HBcAb in serum is indicative of previous or current HBV infections. Consistent with our previous findings, our results confirmed that the rate of circulating anti-HBcAb was also lower in patients with SLE compared to the control group. These analyses therefore suggested that SLE autoimmunity exerted a protection against HBV infections. The mechanism of lower prevalence of HBV in SLE patients is still unclear. One explanation is that cytokines including IFN-α, IL-6, TNF-α are over-expressed in SLE patients. IFN-α can induce transcription of IFN responsive genes through Jak-STATs signaling pathway, which plays a critical role in the innate immune response against viral infections [36]. IFN-α is commonly used for the treatment of chronic hepatitis B (CHB) patients [37]. IL-6 production is also increased in SLE patients and involved in lupus pathogenesis [38]. Interestingly, IL-6 is a key cytokine for early control HBV infection through suppression of HBV transcription, antigen expression and replication [39]. Therefore enhanced production of IL-6 in SLE patients may have an inhibitory effect on HBV infection.

Another possibility is the association of androgen deficiency observed in SLE with HBV infection [40]. As two large prospective studies have shown, HBV carriers exhibited higher androgen concentrations than healthy controls [41, 42], therefore androgen deficiency observed in SLE patients may prevent the establishment of chronic HBV infection.

Unlike HBV, this meta-analysis demonstrated that the prevalence of HCV in SLE patients was higher than in the control group. HCV and HBV belong to different viral families (Flaviviridae and Hepadnoviridae, respectively). They are characterized by different genetic structures and epidemic features. At present, the reason for the high prevalence of HCV in SLE patients remains unclear. One explanation for higher frequency of HCV infection in patients with SLE compared to the control population could be attributed immunological dysregulation observed among SLE patients, and this may be further compounded by immunosuppressive therapies.

The present study has some limitations. First, most of the studies included in these meta-analyses were conducted in Asia where the rates of CHB are high. Additional studies regarding the percentage of HBV infection among SLE patients in other regions, such as Europe and America, should be performed in the future. Second, several studies relied on HCV diagnosis using only a traditional Elisa approach, rather that HCV RNA analysis, which is a more sensitive and accurate detection method. Nevertheless, to our knowledge, this is the first comprehensive meta-analysis to evaluate up-to-date observational studies that reported a relationship between SLE and HBV or HCV infections.

In conclusion, this meta-analysis revealed a relationship between HBV/HCV infection and SLE. Interestingly, we identified a low prevalence of HBV infection and a high prevalence of HCV infection in patients with SLE. However, the precise mechanism by which this occurs remains obscure. Further mechanistic studies are necessary to determine the role of SLE in HBV/HCV infection.

## MATERIALS AND METHODS

### Search strategy

The following databases were used in this study: PubMed/MEDLINE, EMBASE, as well as two major Chinese databases, China National Knowledge Infrastructure (CNKI) and Wanfang Data. The keywords or subject headings used were ‘‘hepatitis B or HBV or HBsAg or HBcAb or Hepatitis C or HCV’’ and ‘‘systemic lupus erythematosus or SLE’’, from the earliest date available to June 30, 2017. Non-English publications were also included. The bibliographies of selected original studies, reviews, and relevant abstracts were screened.

### Inclusion and exclusion criteria

For each eligible study, the title and/or abstract were screened independently by two authors. Any disagreement was resolved by discussion between the authors. We included studies that met the following inclusion criteria: (1) an original case–control study (retrospective, prospective) comparing HBV or HCV infection rate between SLE patients and controls; (2) study reporting the absolute numbers of cases and controls as well as the positive rate of HBV or HCV infection; (3) Described the serological markers for defined HBV and HCV infection. Abstracts, reviews, letters, case reports, and studies that did not provide sufficient data were excluded.

### Quality assessment

The methodological quality of the included studies was assessed by New castle Ottawa Scale (NOS). NOS, a star system allowing a semi-quantitative assessment of nonrandomized study quality for observed studies, contained eight items that were categorized into three major components, including selection, comparability, and exposure (case-control studies). NOS assigns a maximum of 4 points for selection, 2 for comparability, and 3 for exposure or outcome. We assigned NOS scores of 1–3, 4–6, and 7–9 for low-, intermediate-, and high-quality studies, respectively.

### Data extraction

We obtained the following data from each article by using a standardized protocol: last name of the first author, study name, publication year, country where the study was conducted, prospective or retrospective nature of the study, diagnostic method of HBV infection, number of patients with HBV or HCV infection in SLE and control groups as well as the details of the controls used in the studies.

### Statistical analysis

The meta-analysis was performed using Review Manager 5.3 software from the Cochrane Collaboration (London, UK). We calculated the odds ratio (OR) and 95% confidence intervals (CI) to assess the prevalence of HBV and HCV infection in the SLE group compared with the control group. In the forest plots, OR > 1 represented a risk effect and OR < 1 indicated a protective effect. The heterogeneity of the studies was evaluated using the Chi-square test and *I^2^* statistic. The fixed-effects model was used if the heterogeneity was insignificant (Chi-square test *P* ≥ 0.10 and *I^2^* ≤ 50%), while the random-effects model was preferred if significant heterogeneity was found (Chi-square test *P* < 0.10 or *I^2^* > 50%). The funnel plot and Egger's test were used to analyze publication bias.

## SUPPLEMENTARY MATERIALS FIGURES AND TABLES


