# Supplementary material for: Prevalence of hepatitis B virus and hepatitis C virus infection in patients with systemic lupus erythematosus: a systematic review and meta-analysis
Source: Oncotarget. 2017 Nov 1;8(60):102437–45. doi: 10.18632/oncotarget.22261 (PMC5731969; doi:10.18632/oncotarget.22261)
Supplement: Supplementary file 1 [file oncotarget-08-102437-s001.pdf]

Prevalence of hepatitis B virus and hepatitis C virus infection in patients with systemic lupus erythematosus: a systematic review and meta-analysis

SUPPLEMENTARY MATERIALS

Study or Subgroup	SLE Events	SLE Total	Control Events	Control Total	Weight	Odds Ratio M-H, Fixed, 95% CI
**4.2.1 PCR group**						
Costa, C.A., et al. 2002	6	91	123	8867	4.6%	5.02 [2.15, 11.70]
Feng, H., et al. 2006	13	92	2	58	4.2%	4.61 [1.00, 21.23]
Mercado, U., et al. 2005	1	110	3	300	3.1%	0.91 [0.09, 8.82]
Mowla, K. and E. Hajiani, 2008	3	109	1	110	1.9%	3.08 [0.32, 30.13]
Ramos-Casals, M., et al. 2000	15	134	2	200	2.8%	12.48 [2.80, 55.53]
**Subtotal (95% CI)**		**536**		**9535**	**16.6%**	**5.18 [2.74, 9.80]**
Total events	38		131			
Heterogeneity: Chi² = 3.81, df = 4 (P = 0.43); I² = 0%						
Test for overall effect: Z = 5.05 (P < 0.00001)						
**4.2.2 Non-PCR group**						
Agmon-Levin, N., et al. 2009	2	108	1	236	1.2%	4.43 [0.40, 49.44]
Berkun, Y., et al. 2009	1	120	1	140	1.8%	1.17 [0.07, 18.88]
Mahroum, N., et al.2017	53	5018	98	25090	63.8%	2.72 [1.95, 3.81]
Wang, S., et al. 2016	10	844	11	1348	16.5%	1.46 [0.62, 3.45]
**Subtotal (95% CI)**		**6090**		**26814**	**83.4%**	**2.46 [1.80, 3.36]**
Total events	66		111			
Heterogeneity: Chi² = 2.27, df = 3 (P = 0.52); I² = 0%						
Test for overall effect: Z = 5.68 (P < 0.00001)						
**Total (95% CI)**		**6626**		**36349**	**100.0%**	**2.91 [2.21, 3.84]**
Total events	104		242			
Heterogeneity: Chi² = 9.77, df = 8 (P = 0.28); I² = 18%						
Test for overall effect: Z = 7.63 (P < 0.00001)						
Test for subgroup differences: Chi² = 4.21, df = 1 (P = 0.04), I² = 76.3%						

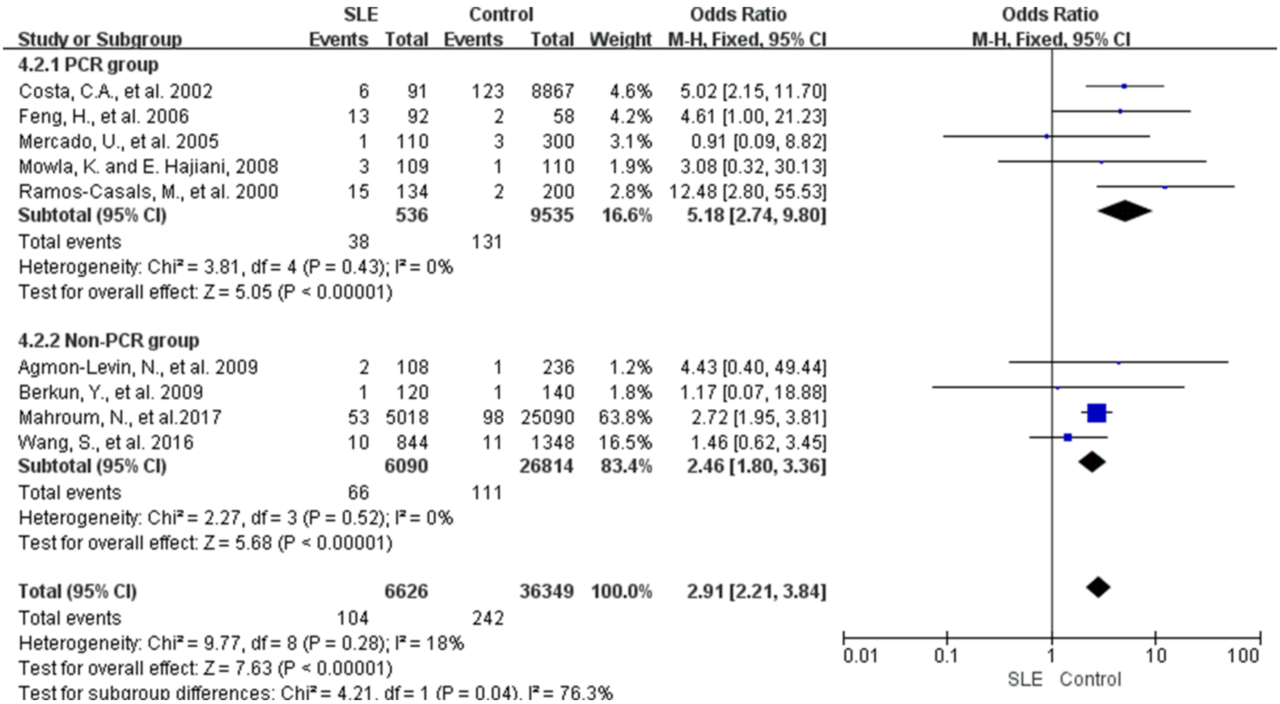

Supplementary Figure 1: Subgroup analysis of the prevalence rate for HCV in SLE patients and controls. Forest plot shows the prevalence rate for HCV in SLE patients and controls, OR, odds ratio; CI, confidence Interval.

Supplementary Table 1: Sensitivity analysis of HBcAb positive rate in SLE patients by omitting each study at a time

Excluded Study	OR (95% Cl)	I^2	P
Wang F, et al. 2016	0.40 (0.31–0.50)	0%	0.56
Watanabe R, et al. 2014	0.29 (0.19–0.44)	50%	0.08
Ram M, et al. 2008	0.33 (0.23–0.49)	59%	0.03
Berkun Y, et al. 2009	0.33 (0.23–0.48)	59%	0.03
Lu CL, et al. 1997	0.32 (0.20–0.51)	60%	0.03
Wu CX, et al. 2015	0.29 (0.19–0.45)	54%	0.06
Qiu N, et al. 2001	0.31 (0.21–0.47)	60%	0.03

Supplementary Table 2: Sentitivity analysis of HCV positive rate in SLE patients by omitting each study at a time

Excluded Study	OR (95% Cl)	I^2	P
Feng H, et al. 2006	2.84 (2.15–3.76)	25%	0.23
Mahroum N, et al. 2017	3.25 (2.02–5.25)	26%	0.22
Ramos-Casals M, et al. 2000	2.64 (1.98–3.51)	0%	0.54
Agmon-Levin N, et al. 2009	2.90 (2.20–3.82)	27%	0.21
Mercado U, et al. 2005	2.98 (2.26–3.93)	20%	0.27
Wang S, et al. 2016	3.20 (2.40–4.28)	2%	0.41
Berkun Y, et al. 2009	2.95 (2.24–3.88)	25%	0.23
Mowla K. and E. Hajiani, 2008	2.91 (2.21–3.84)	28%	0.20
Costa CA, et al. 2002	2.81 (2.10–3.75)	25%	0.24
